# (*R*)-2′-Benz­yloxy-5,5′,6,6′,7,7′,8,8′-octa­hydro-1,1′-binaphthyl-2-ol

**DOI:** 10.1107/S1600536810002308

**Published:** 2010-01-23

**Authors:** Artur R. Abreu, Manuela Ramos Silva, Mariette M. Pereira, J. Carles Bayon, Ana Matos Beja

**Affiliations:** a Chemistry Department, University of Coimbra, P-3004-516 Coimbra, Portugal; b CEMDRX, Physics Department, University of Coimbra, P-3004-516 Coimbra, Portugal; c Chemistry Department, University of Barcelona, Bellaterra, 08193 Barcelona, Portugal

## Abstract

The mol­ecules of the title compound, C_27_H_28_O_2_, exhibit axial chirality. The planes of the aromatic rings of the tetra­lin ring systems make an angle of 85.72 (11)°. The non-aromatic rings adopt distorted half-chair conformations. In one of them, two C atoms of the four-atom aliphatic chain are disordered over two sites in a 0.75 (2):0.25 (2) ratio. The substituent phenyl ring is also disordered over two positions in a 0.59 (3):0.41 (3) ratio. There are no conventional hydrogen bonds joining the mol­ecules.

## Related literature

For the use of 1,1′-binaphthyl-2,2′-diol in asymmetric synthesis, see: Brunel (2005 ▶) Nájera *et al.* (2009 ▶). For the catalytic properties of related compounds, see: Zhang *et al.* (1997 ▶); Reetz *et al.* (1997 ▶); Chan *et al.* (1997 ▶); Waltz *et al.* (2004 ▶). For the synthetic procedure, see: Carrilho *et al.* (2009 ▶); Abreu *et al.* (2010 ▶).
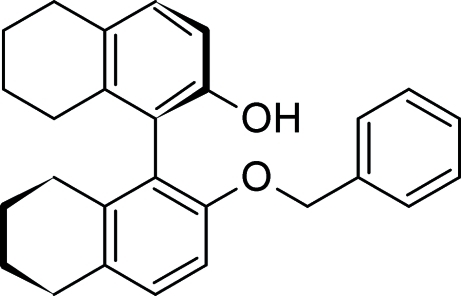


## Experimental

### 

#### Crystal data

C_27_H_28_O_2_
*M* *_r_* = 384.49
Orthorhombic, 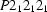

*a* = 8.9871 (3) Å
*b* = 11.6926 (3) Å
*c* = 20.0324 (5) Å
*V* = 2105.06 (10) Å^3^
*Z* = 4
Mo *K*α radiation
μ = 0.08 mm^−1^
*T* = 293 K
0.30 × 0.30 × 0.22 mm

#### Data collection

Bruker SMART APEX CCD area-detector diffractometer
Absorption correction: multi-scan (*SADABS*; Sheldrick, 2000) *T*_min_ = 0.944, *T*_max_ = 0.999
26201 measured reflections
2294 independent reflections
1798 reflections with *I* > 2σ(*I*)
*R*_int_ = 0.026

#### Refinement

*R*[*F* ^2^ > 2σ(*F* ^2^)] = 0.038
*wR*(*F* ^2^) = 0.094
*S* = 1.09
2294 reflections
329 parameters
H-atom parameters constrained
Δρ_max_ = 0.12 e Å^−3^
Δρ_min_ = −0.15 e Å^−3^

### 

Data collection: *SMART* (Bruker, 2003 ▶); cell refinement: *SAINT* (Bruker, 2003 ▶); data reduction: *SAINT*; program(s) used to solve structure: *SHELXS97* (Sheldrick, 2008 ▶); program(s) used to refine structure: *SHELXL97* (Sheldrick, 2008 ▶); molecular graphics: *ORTEPII* (Johnson, 1976 ▶); software used to prepare material for publication: *SHELXL97*.

## Supplementary Material

Crystal structure: contains datablocks global, I. DOI: 10.1107/S1600536810002308/om2312sup1.cif

Structure factors: contains datablocks I. DOI: 10.1107/S1600536810002308/om2312Isup2.hkl

Additional supplementary materials:  crystallographic information; 3D view; checkCIF report

## Figures and Tables

**Fig. 1.**
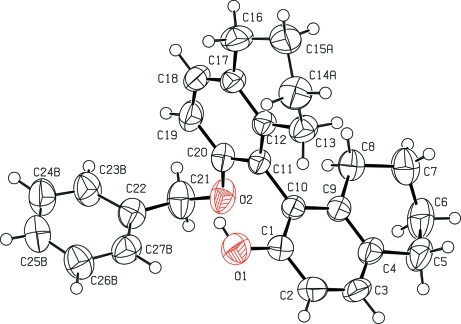
ORTEPII (Johnson, 1976) plot of the title compound. Displacement ellipsoids are drawn at the 50% level. For clarity reasons, only one of the disordered positions is shown.

## supplementary crystallographic information

### Comment

Over the last twenty years an explosive growth of the research in the field of asymmetric synthesis has occurred. The aim of such enantioselective synthesis is to produce chiral optically pure products. Chiral catalysts are often used to promote reactions and lead to the formation of enantiomericaly pure or enriched produts. 1,1'-binaphthyl-2,2'-diol (BINOL) and its derivatives are some of the most successful chiral catalysts in asymmetric synthesis (Brunel, 2005, Nájera *et al.*, 2009). Catalysts containing partially hydrogenated BINOL ligands, 5,5',6,6',7,7',8,8'-octahydro-1,1'-bi-2-naphthol (H_8_-BINOL) and 5,6,7,8-tetrahydro1,1'-bi-2-naphthol (H_4_-BINOL) very often exhibited better stereoselectivity than those obtained from the corresponding BINOL catalysts (Zhang *et al.*, 1997, Reetz *et al.*, 1997, Chan *et al.*, 1997, Waltz *et al.* 2004) Within our project of synthesizing BINOL and H_8_-BINOL derivatives (Carrilho *et al.*, 2009, Abreu *et al.*, 2010), we have obtained the title compound, C_27_H_28_O_2_. The molecules of the title compound, that lack a chiral carbon center, exhibit axial chirality: due to a restrited rotation around the aryl-aryl bond, the tetralin rings have a spatial arrangement that is not superposable on its mirror image. The angle between the planes of the aromatic rings of the tetralin ring systems is 85.72 (11)°. The C1—C10—C11—C20 torsion angle is -95.4 (3). In one of the fused ring systems, the four-atom aliphatic chain is disordered over two sites in a 0.75:0.25 ratio. The phenyl ring of the benzyloxy group is also disordered over two close positions (0.60:0.40). The non-aromatic rings adopt distorted half-chair conformations. There are no conventional hydrogen bonds joining the molecules, the H atom attached to O1 points to the π cloud of the aromatic ring C11/C12/C17/C18/C19/C20 with a distance to the centroid of 3.10° A and an O—H···centroid angle of 141.4°.

### Experimental

The title compound was synthesized according to the previously reported method (Carrilho *et al.*, 2009, Abreu *et al.*, 2010).

### Refinement

Due to the absence of a strong anomalous scatterer, Friedel pairs were merged.

H-atoms were positioned geometrically and refined using a riding model, with C—H = 0.93 Å (aromatic H) and *U*_iso_(H) = 1.2*U*_eq_(C), with C—H = 0.97 Å (CH_2_) and *U*_iso_(H) = 1.2*U*_eq_(C). O—H distance was set to 0.82 Å and *U*_iso_(H) = 1.5*U*_eq_(O). but the torsion angle was refined to fit the electron density.

The title compound shows static disorder and it was necessary to divide many atoms in two partitions. Atoms C23, C24, C25, C26, C27 were refined as disordered over two partially occupied positions, with an occupancy ratio of 0.41 (3) and 0.59 (3). SIMU restraints were used to relate the displacement factor of both partitions. Atoms C14 and C15 were also refined as disordered over two partially occupied positions with occupancy ratio of 0.75 (2) and 0.25 (2).

### Crystal data

**Table:**

C_27_H_28_O_2_	*F*(000) = 824
*M**_r_* = 384.49	*D*_x_ = 1.213 Mg m^−^^3^
Orthorhombic, *P*2_1_2_1_2_1_	Mo *K*α radiation, λ = 0.71073 Å
Hall symbol: P 2ac 2ab	Cell parameters from 5906 reflections
*a* = 8.9871 (3) Å	θ = 2.5–22.4°
*b* = 11.6926 (3) Å	µ = 0.08 mm^−^^1^
*c* = 20.0324 (5) Å	*T* = 293 K
*V* = 2105.06 (10) Å^3^	Prism, colorless
*Z* = 4	0.30 × 0.30 × 0.22 mm

### Data collection

**Table:**

Bruker SMART APEX CCD area-detector diffractometer	2294 independent reflections
Radiation source: fine-focus sealed tube	1798 reflections with *I* > 2σ(*I*)
graphite	*R*_int_ = 0.026
φ and ω scans	θ_max_ = 25.8°, θ_min_ = 2.0°
Absorption correction: multi-scan (*SADABS*; Sheldrick, 2000)	*h* = −10→10
*T*_min_ = 0.944, *T*_max_ = 0.999	*k* = −13→14
26201 measured reflections	*l* = −24→24

### Refinement

**Table:**

Refinement on *F*^2^	Secondary atom site location: difference Fourier map
Least-squares matrix: full	Hydrogen site location: inferred from neighbouring sites
*R*[*F*^2^ > 2σ(*F*^2^)] = 0.038	H-atom parameters constrained
*wR*(*F*^2^) = 0.094	*w* = 1/[σ^2^(*F*_o_^2^) + (0.0428*P*)^2^ + 0.2705*P*] where *P* = (*F*_o_^2^ + 2*F*_c_^2^)/3
*S* = 1.09	(Δ/σ)_max_ < 0.001
2294 reflections	Δρ_max_ = 0.12 e Å^−^^3^
329 parameters	Δρ_min_ = −0.15 e Å^−^^3^
Primary atom site location: structure-invariant direct methods	

### Special details

**Table:**

Geometry. All e.s.d.'s (except the e.s.d. in the dihedral angle between two l.s. planes) are estimated using the full covariance matrix. The cell e.s.d.'s are taken into account individually in the estimation of e.s.d.'s in distances, angles and torsion angles; correlations between e.s.d.'s in cell parameters are only used when they are defined by crystal symmetry. An approximate (isotropic) treatment of cell e.s.d.'s is used for estimating e.s.d.'s involving l.s. planes.
Refinement. Refinement of *F*^2^ against ALL reflections. The weighted *R*-factor *wR* and goodness of fit *S* are based on *F*^2^, conventional *R*-factors *R* are based on *F*, with *F* set to zero for negative *F*^2^. The threshold expression of *F*^2^ > σ(*F*^2^) is used only for calculating *R*-factors(gt) *etc*. and is not relevant to the choice of reflections for refinement. *R*-factors based on *F*^2^ are statistically about twice as large as those based on *F*, and *R*- factors based on ALL data will be even larger.

### Fractional atomic coordinates and isotropic or equivalent isotropic displacement parameters (Å^2^)

**Table:**

	*x*	*y*	*z*	*U*_iso_*/*U*_eq_	Occ. (<1)
O1	0.5068 (3)	0.40183 (17)	0.26056 (9)	0.0769 (6)	
H1	0.4498	0.4536	0.2500	0.115*	
O2	0.1397 (2)	0.34165 (15)	0.17560 (10)	0.0679 (5)	
C1	0.5140 (3)	0.32318 (19)	0.20970 (12)	0.0529 (6)	
C2	0.6048 (3)	0.2290 (2)	0.21946 (13)	0.0622 (7)	
H2	0.6582	0.2207	0.2589	0.075*	
C3	0.6146 (3)	0.1486 (2)	0.17029 (13)	0.0604 (7)	
H3	0.6764	0.0858	0.1768	0.072*	
C4	0.5357 (3)	0.15730 (18)	0.11101 (12)	0.0522 (6)	
C5	0.5454 (4)	0.0637 (2)	0.05917 (16)	0.0709 (8)	
H5A	0.6390	0.0710	0.0354	0.085*	
H5B	0.5454	−0.0098	0.0816	0.085*	
C6	0.4207 (4)	0.0661 (3)	0.00973 (16)	0.0836 (10)	
H6A	0.4431	0.0143	−0.0267	0.100*	
H6B	0.3300	0.0400	0.0311	0.100*	
C7	0.3975 (4)	0.1834 (3)	−0.01718 (14)	0.0765 (9)	
H7A	0.3179	0.1817	−0.0499	0.092*	
H7B	0.4874	0.2085	−0.0396	0.092*	
C8	0.3580 (3)	0.2683 (2)	0.03772 (12)	0.0589 (7)	
H8A	0.3731	0.3452	0.0209	0.071*	
H8B	0.2533	0.2602	0.0484	0.071*	
C9	0.4471 (3)	0.25388 (18)	0.10066 (11)	0.0443 (5)	
C10	0.4371 (3)	0.33731 (18)	0.15065 (10)	0.0430 (5)	
C11	0.3478 (3)	0.44389 (19)	0.14014 (10)	0.0436 (5)	
C12	0.4165 (3)	0.5432 (2)	0.11576 (11)	0.0450 (6)	
C13	0.5816 (3)	0.5420 (2)	0.10123 (14)	0.0579 (7)	
H13A	0.5986	0.4991	0.0605	0.069*	
H13B	0.6322	0.5023	0.1372	0.069*	
C14A	0.6497 (9)	0.6605 (7)	0.0935 (6)	0.0717 (19)	0.75 (2)
H14A	0.7489	0.6542	0.0748	0.086*	0.75 (2)
H14B	0.6574	0.6971	0.1368	0.086*	0.75 (2)
C15A	0.5503 (9)	0.7326 (5)	0.0470 (5)	0.077 (2)	0.75 (2)
H15A	0.5972	0.8059	0.0385	0.093*	0.75 (2)
H15B	0.5377	0.6935	0.0047	0.093*	0.75 (2)
C23A	−0.112 (4)	0.489 (4)	0.281 (3)	0.069 (4)	0.41 (3)
H23A	−0.1758	0.5157	0.2478	0.083*	0.41 (3)
C24A	−0.112 (3)	0.540 (4)	0.347 (2)	0.080 (4)	0.41 (3)
H24A	−0.1781	0.5976	0.3585	0.096*	0.41 (3)
C25A	−0.009 (3)	0.499 (3)	0.3897 (18)	0.075 (4)	0.41 (3)
H25A	−0.0053	0.5284	0.4327	0.090*	0.41 (3)
C26A	0.090 (4)	0.416 (2)	0.3722 (17)	0.072 (4)	0.41 (3)
H26A	0.1616	0.3944	0.4035	0.086*	0.41 (3)
C27A	0.092 (6)	0.365 (3)	0.316 (3)	0.064 (4)	0.41 (3)
H27A	0.1601	0.3073	0.3057	0.077*	0.41 (3)
C14B	0.635 (3)	0.641 (2)	0.0616 (14)	0.067 (5)	0.25 (2)
H14C	0.7425	0.6449	0.0642	0.081*	0.25 (2)
H14D	0.6077	0.6301	0.0152	0.081*	0.25 (2)
C15B	0.571 (2)	0.7482 (16)	0.0862 (18)	0.078 (6)	0.25 (2)
H15C	0.6133	0.8116	0.0616	0.093*	0.25 (2)
H15D	0.5964	0.7578	0.1329	0.093*	0.25 (2)
C23B	−0.142 (3)	0.469 (3)	0.2812 (17)	0.069 (4)	0.59 (3)
H23B	−0.2175	0.4721	0.2495	0.082*	0.59 (3)
C24B	−0.157 (2)	0.532 (2)	0.3375 (13)	0.080 (4)	0.59 (3)
H24B	−0.2376	0.5810	0.3422	0.095*	0.59 (3)
C25B	−0.052 (2)	0.5232 (18)	0.3887 (11)	0.074 (4)	0.59 (3)
H25B	−0.0627	0.5666	0.4274	0.089*	0.59 (3)
C26B	0.065 (2)	0.4502 (18)	0.3814 (11)	0.071 (4)	0.59 (3)
H26B	0.1335	0.4389	0.4157	0.086*	0.59 (3)
C27B	0.079 (4)	0.392 (2)	0.319 (2)	0.064 (4)	0.59 (3)
H27B	0.1612	0.3440	0.3128	0.077*	0.59 (3)
C16	0.4025 (4)	0.7508 (2)	0.07886 (14)	0.0699 (8)	
H16A	0.3358	0.7850	0.0465	0.084*	
H16B	0.4138	0.8044	0.1155	0.084*	
C17	0.3329 (3)	0.6418 (2)	0.10512 (11)	0.0509 (6)	
C18	0.1833 (3)	0.6394 (2)	0.12075 (12)	0.0599 (7)	
H18	0.1270	0.7052	0.1143	0.072*	
C19	0.1144 (3)	0.5433 (2)	0.14556 (12)	0.0600 (7)	
H19	0.0137	0.5448	0.1563	0.072*	
C20	0.1962 (3)	0.4446 (2)	0.15439 (11)	0.0505 (6)	
C21	−0.0075 (3)	0.3394 (3)	0.20324 (14)	0.0707 (8)	
H21A	−0.0377	0.2605	0.2095	0.085*	
H21B	−0.0759	0.3742	0.1718	0.085*	
C22	−0.0181 (3)	0.4012 (2)	0.26870 (13)	0.0560 (7)	

### Atomic displacement parameters (Å^2^)

**Table:**

	*U*^11^	*U*^22^	*U*^33^	*U*^12^	*U*^13^	*U*^23^
O1	0.0951 (16)	0.0787 (13)	0.0568 (10)	0.0208 (12)	−0.0111 (11)	−0.0132 (9)
O2	0.0558 (11)	0.0554 (10)	0.0924 (12)	−0.0041 (9)	0.0288 (10)	−0.0081 (10)
C1	0.0592 (16)	0.0494 (13)	0.0500 (13)	0.0042 (13)	0.0045 (12)	0.0043 (11)
C2	0.0661 (18)	0.0623 (16)	0.0583 (14)	0.0106 (15)	0.0012 (14)	0.0150 (13)
C3	0.0609 (17)	0.0441 (13)	0.0763 (16)	0.0138 (13)	0.0138 (14)	0.0197 (13)
C4	0.0524 (15)	0.0348 (11)	0.0696 (15)	−0.0013 (12)	0.0172 (13)	0.0057 (11)
C5	0.078 (2)	0.0419 (14)	0.093 (2)	0.0029 (15)	0.0260 (18)	−0.0077 (14)
C6	0.089 (2)	0.0685 (19)	0.093 (2)	−0.0068 (18)	0.019 (2)	−0.0367 (17)
C7	0.078 (2)	0.085 (2)	0.0667 (16)	−0.0009 (18)	0.0029 (15)	−0.0224 (16)
C8	0.0582 (16)	0.0596 (15)	0.0589 (14)	0.0007 (14)	0.0023 (13)	−0.0099 (12)
C9	0.0427 (13)	0.0364 (11)	0.0539 (12)	−0.0036 (10)	0.0107 (11)	0.0026 (10)
C10	0.0437 (13)	0.0371 (11)	0.0483 (11)	−0.0010 (11)	0.0089 (11)	0.0043 (10)
C11	0.0476 (14)	0.0399 (12)	0.0434 (11)	0.0064 (11)	0.0010 (10)	−0.0041 (10)
C12	0.0510 (14)	0.0403 (12)	0.0438 (11)	0.0036 (11)	−0.0008 (11)	−0.0030 (10)
C13	0.0558 (17)	0.0485 (14)	0.0694 (15)	0.0006 (13)	0.0048 (14)	0.0055 (13)
C14A	0.069 (4)	0.058 (3)	0.088 (5)	−0.017 (3)	0.006 (4)	0.001 (4)
C15A	0.116 (5)	0.042 (2)	0.075 (4)	−0.008 (3)	0.023 (4)	0.008 (3)
C23A	0.044 (10)	0.093 (10)	0.069 (2)	0.004 (7)	0.010 (7)	−0.002 (6)
C24A	0.069 (10)	0.096 (5)	0.074 (7)	0.012 (9)	0.011 (8)	−0.014 (4)
C25A	0.071 (11)	0.083 (9)	0.069 (2)	−0.002 (6)	0.017 (7)	−0.017 (5)
C26A	0.060 (8)	0.090 (12)	0.065 (6)	−0.007 (7)	0.004 (5)	−0.006 (7)
C27A	0.051 (6)	0.063 (11)	0.080 (4)	0.003 (8)	0.007 (4)	0.008 (9)
C14B	0.065 (9)	0.056 (11)	0.081 (12)	−0.014 (8)	−0.006 (11)	0.007 (10)
C15B	0.088 (11)	0.056 (9)	0.089 (15)	−0.002 (8)	−0.008 (10)	0.010 (10)
C23B	0.044 (9)	0.093 (10)	0.069 (2)	0.003 (6)	0.010 (6)	−0.003 (5)
C24B	0.067 (10)	0.097 (4)	0.074 (7)	0.012 (8)	0.012 (8)	−0.015 (4)
C25B	0.071 (11)	0.083 (9)	0.069 (2)	−0.002 (6)	0.018 (7)	−0.017 (5)
C26B	0.060 (8)	0.091 (12)	0.064 (6)	−0.007 (7)	0.004 (5)	−0.006 (7)
C27B	0.050 (5)	0.062 (11)	0.080 (4)	0.004 (7)	0.007 (4)	0.008 (9)
C16	0.099 (2)	0.0436 (15)	0.0676 (16)	0.0071 (15)	−0.0067 (17)	0.0063 (13)
C17	0.0662 (18)	0.0420 (13)	0.0444 (12)	0.0092 (13)	−0.0044 (12)	−0.0018 (11)
C18	0.072 (2)	0.0526 (15)	0.0549 (14)	0.0236 (15)	−0.0084 (13)	−0.0043 (12)
C19	0.0492 (16)	0.0681 (17)	0.0627 (15)	0.0151 (14)	0.0028 (13)	−0.0103 (14)
C20	0.0492 (15)	0.0491 (13)	0.0531 (13)	0.0027 (12)	0.0057 (11)	−0.0084 (11)
C21	0.0506 (16)	0.0779 (17)	0.0835 (18)	−0.0146 (15)	0.0171 (14)	−0.0169 (16)
C22	0.0414 (15)	0.0584 (15)	0.0683 (17)	−0.0077 (13)	0.0070 (14)	−0.0010 (13)

### Geometric parameters (Å, °)

**Table:**

O1—C1	1.374 (3)	C23A—C22	1.35 (5)
O1—H1	0.8200	C23A—C24A	1.45 (7)
O2—C20	1.374 (3)	C23A—H23A	0.9300
O2—C21	1.434 (3)	C24A—C25A	1.35 (4)
C1—C10	1.380 (3)	C24A—H24A	0.9300
C1—C2	1.384 (3)	C25A—C26A	1.37 (3)
C2—C3	1.365 (4)	C25A—H25A	0.9300
C2—H2	0.9300	C26A—C27A	1.28 (6)
C3—C4	1.387 (4)	C26A—H26A	0.9300
C3—H3	0.9300	C27A—C22	1.43 (6)
C4—C9	1.397 (3)	C27A—H27A	0.9300
C4—C5	1.511 (3)	C14B—C15B	1.47 (5)
C5—C6	1.495 (5)	C14B—H14C	0.9700
C5—H5A	0.9700	C14B—H14D	0.9700
C5—H5B	0.9700	C15B—C16	1.518 (18)
C6—C7	1.488 (4)	C15B—H15C	0.9700
C6—H6A	0.9700	C15B—H15D	0.9700
C6—H6B	0.9700	C23B—C24B	1.35 (4)
C7—C8	1.524 (3)	C23B—C22	1.39 (3)
C7—H7A	0.9700	C23B—H23B	0.9300
C7—H7B	0.9700	C24B—C25B	1.39 (3)
C8—C9	1.503 (3)	C24B—H24B	0.9300
C8—H8A	0.9700	C25B—C26B	1.367 (17)
C8—H8B	0.9700	C25B—H25B	0.9300
C9—C10	1.401 (3)	C26B—C27B	1.43 (4)
C10—C11	1.497 (3)	C26B—H26B	0.9300
C11—C20	1.392 (3)	C27B—C22	1.34 (4)
C11—C12	1.403 (3)	C27B—H27B	0.9300
C12—C17	1.392 (3)	C16—C17	1.515 (4)
C12—C13	1.512 (4)	C16—H16A	0.9700
C13—C14B	1.48 (2)	C16—H16B	0.9700
C13—C14A	1.523 (8)	C17—C18	1.380 (4)
C13—H13A	0.9700	C18—C19	1.376 (4)
C13—H13B	0.9700	C18—H18	0.9300
C14A—C15A	1.542 (17)	C19—C20	1.379 (4)
C14A—H14A	0.9700	C19—H19	0.9300
C14A—H14B	0.9700	C21—C22	1.500 (4)
C15A—C16	1.488 (7)	C21—H21A	0.9700
C15A—H15A	0.9700	C21—H21B	0.9700
C15A—H15B	0.9700		
			
C1—O1—H1	109.5	C24A—C23A—H23A	120.8
C20—O2—C21	118.4 (2)	C25A—C24A—C23A	116 (4)
O1—C1—C10	122.1 (2)	C25A—C24A—H24A	122.1
O1—C1—C2	117.1 (2)	C23A—C24A—H24A	122.1
C10—C1—C2	120.8 (2)	C24A—C25A—C26A	122 (3)
C3—C2—C1	119.0 (2)	C24A—C25A—H25A	118.8
C3—C2—H2	120.5	C26A—C25A—H25A	118.8
C1—C2—H2	120.5	C27A—C26A—C25A	124 (4)
C2—C3—C4	122.3 (2)	C27A—C26A—H26A	117.8
C2—C3—H3	118.8	C25A—C26A—H26A	117.8
C4—C3—H3	118.8	C26A—C27A—C22	116 (3)
C3—C4—C9	118.5 (2)	C26A—C27A—H27A	122.1
C3—C4—C5	120.4 (2)	C22—C27A—H27A	121.0
C9—C4—C5	121.1 (2)	C15B—C14B—C13	111 (2)
C6—C5—C4	113.5 (2)	C15B—C14B—H14C	109.4
C6—C5—H5A	108.9	C13—C14B—H14C	109.4
C4—C5—H5A	108.9	C15B—C14B—H14D	109.4
C6—C5—H5B	108.9	C13—C14B—H14D	109.4
C4—C5—H5B	108.9	H14C—C14B—H14D	108.0
H5A—C5—H5B	107.7	C14B—C15B—C16	112 (2)
C7—C6—C5	111.2 (3)	C14B—C15B—H15C	109.2
C7—C6—H6A	109.4	C16—C15B—H15C	109.2
C5—C6—H6A	109.4	C14B—C15B—H15D	109.2
C7—C6—H6B	109.4	C16—C15B—H15D	109.2
C5—C6—H6B	109.4	H15C—C15B—H15D	107.9
H6A—C6—H6B	108.0	C24B—C23B—C22	123 (2)
C6—C7—C8	111.8 (2)	C24B—C23B—H23B	118.7
C6—C7—H7A	109.3	C22—C23B—H23B	118.7
C8—C7—H7A	109.3	C23B—C24B—C25B	121 (2)
C6—C7—H7B	109.3	C23B—C24B—H24B	119.7
C8—C7—H7B	109.3	C25B—C24B—H24B	119.7
H7A—C7—H7B	107.9	C26B—C25B—C24B	119.2 (19)
C9—C8—C7	114.1 (2)	C26B—C25B—H25B	120.4
C9—C8—H8A	108.7	C24B—C25B—H25B	120.4
C7—C8—H8A	108.7	C25B—C26B—C27B	117 (2)
C9—C8—H8B	108.7	C25B—C26B—H26B	121.3
C7—C8—H8B	108.7	C27B—C26B—H26B	121.3
H8A—C8—H8B	107.6	C22—C27B—C26B	124 (2)
C4—C9—C10	119.6 (2)	C22—C27B—H27B	118.0
C4—C9—C8	121.2 (2)	C26B—C27B—H27B	118.0
C10—C9—C8	119.2 (2)	C15A—C16—C17	113.4 (3)
C1—C10—C9	119.8 (2)	C17—C16—C15B	111.1 (7)
C1—C10—C11	119.25 (19)	C15A—C16—H16A	108.9
C9—C10—C11	120.92 (19)	C17—C16—H16A	108.9
C20—C11—C12	119.8 (2)	C15B—C16—H16A	133.5
C20—C11—C10	120.1 (2)	C15A—C16—H16B	108.9
C12—C11—C10	120.2 (2)	C17—C16—H16B	108.9
C17—C12—C11	120.1 (2)	C15B—C16—H16B	80.6
C17—C12—C13	120.5 (2)	H16A—C16—H16B	107.7
C11—C12—C13	119.4 (2)	C18—C17—C12	118.3 (2)
C14B—C13—C12	114.4 (10)	C18—C17—C16	119.9 (2)
C12—C13—C14A	113.9 (4)	C12—C17—C16	121.8 (2)
C14B—C13—H13A	84.3	C19—C18—C17	122.5 (3)
C12—C13—H13A	108.8	C19—C18—H18	118.8
C14A—C13—H13A	108.8	C17—C18—H18	118.8
C14B—C13—H13B	128.3	C18—C19—C20	119.3 (2)
C12—C13—H13B	108.8	C18—C19—H19	120.3
C14A—C13—H13B	108.8	C20—C19—H19	120.3
H13A—C13—H13B	107.7	O2—C20—C19	125.1 (2)
C13—C14A—C15A	109.0 (8)	O2—C20—C11	114.8 (2)
C13—C14A—H14A	109.9	C19—C20—C11	120.0 (2)
C15A—C14A—H14A	109.9	O2—C21—C22	112.8 (2)
C13—C14A—H14B	109.9	O2—C21—H21A	109.0
C15A—C14A—H14B	109.9	C22—C21—H21A	109.0
H14A—C14A—H14B	108.3	O2—C21—H21B	109.0
C16—C15A—C14A	109.6 (7)	C22—C21—H21B	109.0
C16—C15A—H15A	109.7	H21A—C21—H21B	107.8
C14A—C15A—H15A	109.7	C27B—C22—C23B	115.8 (18)
C16—C15A—H15B	109.7	C23A—C22—C27A	123 (3)
C14A—C15A—H15B	109.7	C27B—C22—C21	125.1 (12)
H15A—C15A—H15B	108.2	C23A—C22—C21	124 (2)
C22—C23A—C24A	118 (4)	C23B—C22—C21	119.0 (14)
C22—C23A—H23A	120.8	C27A—C22—C21	112.8 (19)
			
O1—C1—C2—C3	179.7 (2)	C12—C13—C14B—C15B	44 (3)
C10—C1—C2—C3	−1.5 (4)	C14A—C13—C14B—C15B	−51 (3)
C1—C2—C3—C4	−0.6 (4)	C13—C14B—C15B—C16	−63 (3)
C2—C3—C4—C9	2.2 (4)	C22—C23B—C24B—C25B	−5(4)
C2—C3—C4—C5	−177.5 (2)	C23B—C24B—C25B—C26B	0(4)
C3—C4—C5—C6	161.4 (3)	C24B—C25B—C26B—C27B	4(4)
C9—C4—C5—C6	−18.3 (4)	C25B—C26B—C27B—C22	−2(4)
C4—C5—C6—C7	48.1 (3)	C14A—C15A—C16—C17	−49.2 (10)
C5—C6—C7—C8	−60.5 (4)	C14B—C15B—C16—C17	48 (3)
C6—C7—C8—C9	41.5 (4)	C11—C12—C17—C18	1.6 (3)
C3—C4—C9—C10	−1.7 (3)	C13—C12—C17—C18	−178.4 (2)
C5—C4—C9—C10	178.0 (2)	C11—C12—C17—C16	180.0 (2)
C3—C4—C9—C8	−179.6 (2)	C13—C12—C17—C16	0.0 (4)
C5—C4—C9—C8	0.1 (3)	C15A—C16—C17—C18	−164.1 (6)
C7—C8—C9—C4	−11.7 (3)	C15B—C16—C17—C18	161.5 (15)
C7—C8—C9—C10	170.4 (2)	C15A—C16—C17—C12	17.6 (6)
O1—C1—C10—C9	−179.3 (2)	C15B—C16—C17—C12	−16.8 (15)
C2—C1—C10—C9	1.9 (4)	C12—C17—C18—C19	−0.8 (4)
O1—C1—C10—C11	2.7 (4)	C16—C17—C18—C19	−179.2 (2)
C2—C1—C10—C11	−176.1 (2)	C17—C18—C19—C20	−1.0 (4)
C4—C9—C10—C1	−0.3 (3)	C21—O2—C20—C19	−12.9 (4)
C8—C9—C10—C1	177.7 (2)	C21—O2—C20—C11	168.4 (2)
C4—C9—C10—C11	177.6 (2)	C18—C19—C20—O2	−176.7 (2)
C8—C9—C10—C11	−4.4 (3)	C18—C19—C20—C11	1.9 (4)
C1—C10—C11—C20	−95.4 (3)	C12—C11—C20—O2	177.69 (19)
C9—C10—C11—C20	86.7 (3)	C10—C11—C20—O2	−2.1 (3)
C1—C10—C11—C12	84.8 (3)	C12—C11—C20—C19	−1.0 (3)
C9—C10—C11—C12	−93.2 (3)	C10—C11—C20—C19	179.1 (2)
C20—C11—C12—C17	−0.7 (3)	C20—O2—C21—C22	−67.3 (3)
C10—C11—C12—C17	179.08 (19)	C26B—C27B—C22—C23B	−3(4)
C20—C11—C12—C13	179.3 (2)	C26B—C27B—C22—C21	−178.9 (18)
C10—C11—C12—C13	−0.9 (3)	C24A—C23A—C22—C27A	−6(5)
C17—C12—C13—C14B	−13.4 (14)	C24A—C23A—C22—C21	179 (2)
C11—C12—C13—C14B	166.5 (13)	C24B—C23B—C22—C27B	7(3)
C17—C12—C13—C14A	15.8 (6)	C24B—C23B—C22—C21	−177 (2)
C11—C12—C13—C14A	−164.2 (5)	C26A—C27A—C22—C23A	4(6)
C12—C13—C14A—C15A	−47.0 (9)	C26A—C27A—C22—C21	179 (3)
C13—C14A—C15A—C16	64.6 (11)	O2—C21—C22—C27B	−44.8 (19)
C22—C23A—C24A—C25A	4(5)	O2—C21—C22—C23A	123.0 (19)
C23A—C24A—C25A—C26A	1(6)	O2—C21—C22—C23B	139.0 (12)
C24A—C25A—C26A—C27A	−4(7)	O2—C21—C22—C27A	−52 (2)
C25A—C26A—C27A—C22	1(7)		
